# The crosstalk between insulin resistance and tau pathology

**DOI:** 10.1002/alz.092703

**Published:** 2025-01-03

**Authors:** Rashi Kumar, Ritu Varshney, Sharad Gupta

**Affiliations:** ^1^ Indian Institute of Technology, Gandhinagar India

## Abstract

**Background:**

Diabetes is a modifiable risk factor for Alzheimer’s disease, and GLUT4, an insulin‐dependent transporter, plays a crucial role in insulin‐resistant conditions and, consequently, in diabetes development. The study aimed to investigate the relationship between tau pathology and insulin resistance by quantifying GLUT4 expression and glucose concentration.

**Method:**

Initially, SH‐SY5Y cells underwent transfection with either a wild‐type tau plasmid or a mutant tau plasmid. Subsequently, insulin resistance was induced using high glucose or dexamethasone. The impact of these manipulations was assessed through a glucose uptake assay measuring cellular glucose concentration. Immunocytochemistry techniques were then applied to evaluate GLUT4 expression.

**Result:**

A significant increase in glucose concentration was observed under the latter condition. Additionally, there was a marked decrease in GLUT4 expression in neuronal cells transfected with the mutant tau plasmid, simulating tau pathology.

**Conclusion:**

The study provides evidence supporting insulin resistance as a contributing risk factor for tau pathology development, potentially leading to Alzheimer’s disease later in life. Tau aggregates may increase the likelihood of insulin resistance by impairing the insulin signaling pathway, ultimately resulting in Type 2 diabetes. The findings suggest that impaired insulin signaling in the brain could contribute to tau pathogenesis by decreasing GLUT4 expression, leading to hyperglycemia and cellular hypertrophy.

